# Prediction of Epileptic Seizure by Analysing Time Series EEG Signal Using *k*-NN Classifier

**DOI:** 10.1155/2017/6848014

**Published:** 2017-08-13

**Authors:** Md. Kamrul Hasan, Md. Asif Ahamed, Mohiuddin Ahmad, M. A. Rashid

**Affiliations:** ^1^Department of EEE, Khulna University of Engineering & Technology (KUET), Khulna 9203, Bangladesh; ^2^FSTK, University Sultan Zainal Abidin (UniSZA), 21300 Kuala Terengganu, Terengganu, Malaysia

## Abstract

Electroencephalographic signal is a representative signal that contains information about brain activity, which is used for the detection of epilepsy since epileptic seizures are caused by a disturbance in the electrophysiological activity of the brain. The prediction of epileptic seizure usually requires a detailed and experienced analysis of EEG. In this paper, we have introduced a statistical analysis of EEG signal that is capable of recognizing epileptic seizure with a high degree of accuracy and helps to provide automatic detection of epileptic seizure for different ages of epilepsy. To accomplish the target research, we extract various epileptic features namely approximate entropy (ApEn), standard deviation (SD), standard error (SE), modified mean absolute value (MMAV), roll-off (*R*), and zero crossing (ZC) from the epileptic signal. The *k*-nearest neighbours (*k*-NN) algorithm is used for the classification of epilepsy then regression analysis is used for the prediction of the epilepsy level at different ages of the patients. Using the statistical parameters and regression analysis, a prototype mathematical model is proposed which helps to find the epileptic randomness with respect to the age of different subjects. The accuracy of this prototype equation depends on proper analysis of the dynamic information from the epileptic EEG.

## 1. Introduction

**Figure 1:**
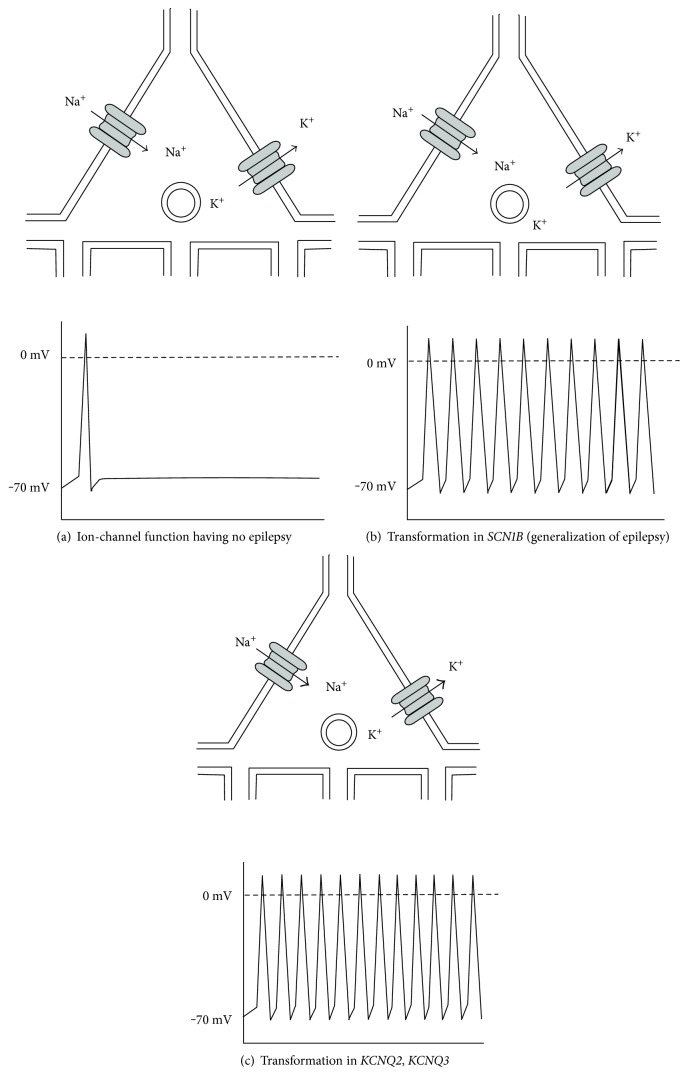
Ion-channel dysfunction for the formation of epilepsy.

Epilepsy is a long-lasting neurological disorder categorized by repeated, gratuitous seizures, electrophysiological disturbances in the human brain which may range from brief gaps of attention or muscle bumps to severe and prolonged seizures. Epileptic seizures are the visible or apparent manifestations that are produced when the brain briefly becomes dysfunctional because of abnormal paroxysmal discharge of the nerve cells in the cerebral cortex [1–3]. Alternately, epilepsy is a group of neurological disorders characterized by epileptic seizures [4, 5]. Epileptic seizures are incidents which may be varied from brief and nearly undetectable to long periods of vigorous shaking [6]. In epilepsy, seizures tend to recur and have no immediate underlying cause while seizures that occur due to a specific cause are not deemed to represent epilepsy [4, 7]. Characteristics of seizures vary and depend on where in the brain the disturbance first starts and how far it spreads. Temporary symptoms occur, such as loss of awareness or consciousness and disturbances of movement, sensation (including vision, hearing, and taste), mood, or other cognitive functions.  Figure 1(a) represents normal neuronal-ion-channel function and in this section the membrane resting potential is −70 mV which is due to the sodium and potassium channels as a primary requirement of action potential.

The sodium and potassium channels are associated with a depolarizing phase which occupy the medium position by sodium channel opening and a repolarizing phase due to potassium-channel opening and sodium-channel inactivation. On the other hand, remaining potassium channels contribute to a longer-term repolarization that acts as the prevention of repetitive excitation of the neuron. In Figure 1(b), mutations in SCN1B, which encode a voltage-gated sodium-channel subunit, are associated with generalized epilepsy with febrile seizures plus [4]. The movement of an increased amount of sodium current, which would lead to a greater depolarization during the action potential and an increased tendency to excite repetitive bursts is the outcome of apparent mutations. Similarly, in Figure 1(c), mutations in KCNQ2 and KCNQ3 will occur in both the potassium and sodium electrodes where encoding of potassium channels occur which are related with benign ancestral neonatal spasms. People with seizures can be injured, have fractures or bruises more frequently than controls, or have higher rates of psychological problems like anxiety or depression which causes more physical problems (such as fractures and bruising from injuries related to seizures). Similarly, the risk of premature death in people with epilepsy is up to 3 times higher than the general population, with the highest rates found in low- and middle-income countries and rural versus urban areas. A great proportion of the causes of death related to epilepsy in low- and middle-income countries are potentially preventable, such as falls, drowning, burns, and prolonged seizures [8–10]. There are more than 30 different forms of epilepsy and more than 40 different types of seizures [2]. According to a report of the World Health Organization (WHO) [11], around 50 million people worldwide have epilepsy. Around 90% of them are from developing countries and one-fourth of them do not have access to medication. Epilepsy cannot be cured, but it can usually be controllable with medication. For initial treatment of epilepsy, antiepileptic drugs (AEDs) are used [12]. Epilepsy is not transmissible. The idiopathic epilepsy is the most common type of epilepsy, which may affect 6 (out of 10) people with the disorder, and it has no detectible cause. Epilepsy which may take place due to known cause is called secondary epilepsy or symptomatic epilepsy. The major causes of secondary epilepsy [11] might be as follows:

- The brain may get impairment from injuries
- Inherited abnormalities with associated brain defects
- A severe head injury
- Stroke may limit the amount of oxygen to the brain
- Some infection like meningitis and encephalitis of the human brain
- A brain tumour which creates more randomness.

There are several methods to diagnose epilepsy such as electroencephalography (EEG), magnetic resonance imaging (MRI), functional magnetic resonance imaging (fMRI), single-photon emission computed tomography (SPECT), positron emission tomography (PET), and magnetoencephalography (MEG). As EEG has speed, high time resolution, and non-invasive advantages, still now it remains one of the most useful and effective tools in the treatment of epilepsy. Prediction of epileptic seizure based on EEG signals can be separated into three classes: time domain, frequency domain, and the nonlinear methods [13]. In recent times, seizure is detected from the recorded seizures in order to quantify the clinical image and propose video-based seizure recognition. In some papers, information-based measure are also proposed for the detections of epileptic seizure [14]. Entropy is a measure of rate of information that may be used in the signal processing for the detection of noise where a higher value corresponds to increased unpredictability while a lower value corresponds to higher predictability [15]. In our proposed research, we use six features for the classification, and among these features, entropy has the higher ranked features that is used for the regression model for prediction of level of epilepsy.

## 2. Mathematical Background of Classifier and Statistical Features

Mathematical background for the classifier (*k*-NN) and statistical features (approximate entropy (ApEn), standard deviation (SD), standard error (SE), modified mean absolute value (MMAV), roll-off (*R*), and zero crossing (ZC)) are described below.

### 2.1. *k*-Nearest Neighbours (*k*-NN)

**Figure 2:**
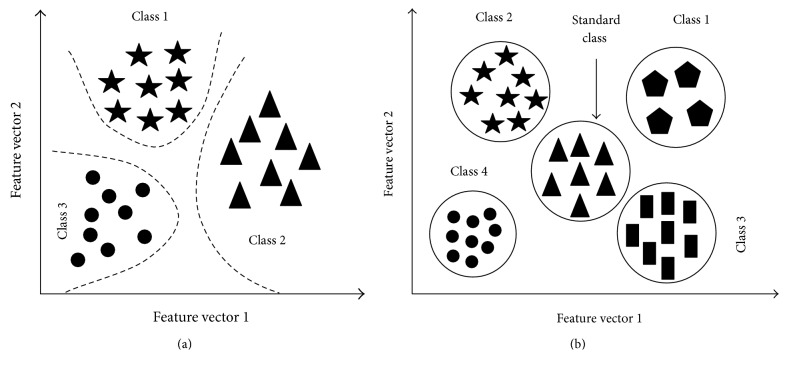
Architecture of *k*-NN classifier (a) simple classification and (b) cluster classification.

The *k*-nearest neighbours (*k*-NN) algorithm is a nonparametric learning algorithm mechanism mainly used for the classification of signal pattern or pattern recognition as shown in Figure 2(a). The major goals of this mechanism are to assign to an unseen point the leading class among its *k*-nearest neighbours within the training sets of data [16, 17].

Among all of the method of classification like support vector machine (SVM), artificial neural network (ANN), linear discriminant analysis (LDA), naive Bayes (NB), and RBF neural network (RBFNN), *k*-NN is the best classifier statistical pattern recognition or neighbour cluster selection as shown in Figure 2(b) due to its consistently high performance, without a priori assumptions. The *k*-NN classifier extends this idea by taking the *k*-nearest points and assigning the sign of the majority [18]. The positive integer “*k*” indicates how many neighbours guide the classification. The default value *k* = 1 is called the nearest neighbour algorithm. In the classification analysis, *k*-NN is the supervised learning algorithm [19, 20]. The learning algorithm of *k*-NN for the classification of any data set *X* is described below step by step.

1. Consider that training categories is the column vector of training set. If there are *i* numbers of categories in a training set which is denoted by  *C*_1_, *C*_2_, *C*_3_,…, *C*_*i*_. The summation makes *m*-dimensional feature vector.
2. The sample data set *X* should have the same dimensional vector for the proper classification which is denoted by *X*_1_, *X*_2_, *X*_3_,…, *X*_*m*_.
3. In this state, the similarity between training set and data set should be calculated. Taking *j*th sample  *d*_*j*_ (*d*_*j*1_, *d*_*j*2_, *d*_*j*3_,…, *d*_*jm*_). The similarity SIM(*X*, *d*_*j*_) is mentioned in

(1) $$\begin{array}{c} SIM\;\left(X,d_{j}\right)=\frac{\sum_{i=1}^{m}\left(X_{i}\times d_{ji}\right)}{\sqrt{{\left(\sum_{i=1}^{m}X_{i}\right)}^{2}{\left(\sum_{i=1}^{m}d_{ji}\right)}^{2}}}. \end{array}$$

(4) Select the value of *k* which is larger from *N* similarity of SIM (*X*, *d*_*i*_) (*i* = 1, 2, 3,…, *N*). Now, the probability function has the following mathematical form:

(2) $$\begin{array}{c} P\;\left(X,C_{i}\right)=\underset{d}{\sum }SIM\left(X,d_{j}\right)\times y\left(d_{j},C_{i}\right), \end{array}$$

where  *y* (*d*_*j*_, *C*_*i*_) is the category of attribute function which satisfies the following mathematics:

(3) $$\begin{array}{c} y\;\left(d_{j},C_{i}\right)=\left\{\begin{array}{cc} 1, & d_{j}\in C_{i}\\ 0, & d_{j}{\notin C}_{i}. \end{array}\right. \end{array}$$

(5) Finally, justification of sample *X* to categories which have larger value of (*X*, *C*_*i*_).

In the *k*-NN classifier, the distance between two sets of data points is measured by some distance vectors, which are Euclidean distance, cityblock distance, cosine distance, and correlation distance.

In statistical mathematics, the Euclidean distance is the distance between two points in Euclidean space, which becomes a metric space whose norm form is commonly known as Euclidean norm. The Euclidean distance, *d*_*st*_, is in

(4) $$\begin{array}{c} d_{st}=\sqrt{\left(x_{s}-y_{t}\right)\times {\left(x_{s}-y_{t}\right)}^{\prime }}. \end{array}$$

The distance between two points is the sum of the absolute differences of their Cartesian coordinates known as the cityblock distance which is also known as Manhattan length [21]. Cityblock distance *d*_*st*_ is represented in

(5) $$\begin{array}{c} d_{st}=\overset{n}{\underset{j=1}{\sum }}\left|x_{sj}-y_{tj}\right|. \end{array}$$

Cosine distance is the distance which is used for the complement in positive space, that is, *D*_*c*_(*A*, *B*) = 1 − *S*_*c*_(*A*, *B*). Cosine distance *d*_*st*_ is represented in

(6) $$\begin{array}{c} d_{st}=\left(1-\frac{x_{s}y_{t}^{\prime }}{\sqrt{\left(x_{s}x_{s}^{\prime }\right)\left(y_{t}y_{t}^{\prime }\right)}}\right). \end{array}$$

Correlation distance is the measure of statistical distance between two random variables or two random vectors of arbitrary, not necessarily equal dimension. Correlation distance *d*_*st*_ is represented in

(7) $$\begin{array}{c} d_{st}=1-\frac{\left(x_{s}-\bar{x_{s}}\;\right){\left(y_{t}-\bar{y_{t}}\;\right)}^{\prime }}{\sqrt{\left(x_{t}\;\bar{x_{s}}\right){\left(x_{t}\;\bar{x_{s}}\right)}^{\prime }}\;\sqrt{\left(y_{t}\;\bar{y_{t}}\right){\left(y_{t}\;\bar{y_{t}}\right)}^{\prime }}}, \end{array}$$

where ${\bar{x}}_{s}=\left(1/n\right)\sum_{j}x_{sj}$ and ${\bar{y}}_{t}=\left(1/n\right)\sum_{j}y_{tj}.$

The statistical features used for the classification using *k*-NN classifier in this research are described below.

### 2.2. Approximate Entropy (ApEn)

ApEn is a statistical feature that indicates the predictability of the current amplitude values of a physiological signal, for example, EEG based on its earlier amplitude. The value of ApEn drops sharply during an epileptic seizure, and this property is used to detect the epileptic seizures. A high value of approximate entropy signifies more irregularity; on the contrary, a low value signifies that the time series is deterministic which reflects the intracortical information flow in the brain when applied to EEG signals [22, 23]. The value of ApEn can be calculated by using

(8) $$\begin{array}{c} \begin{array}{c} {ø}^{m}\left(r\right)={\left(N-m+1\right)}^{-1}\overset{N-m+1}{\underset{i=1}{\sum }}\log\left(c_{i}^{m}\left(r\right)\right),\\ ApEn={ø}^{m}\left(r\right)-{ø}^{m+1}\left(r\right). \end{array} \end{array}$$

**Figure 3:**
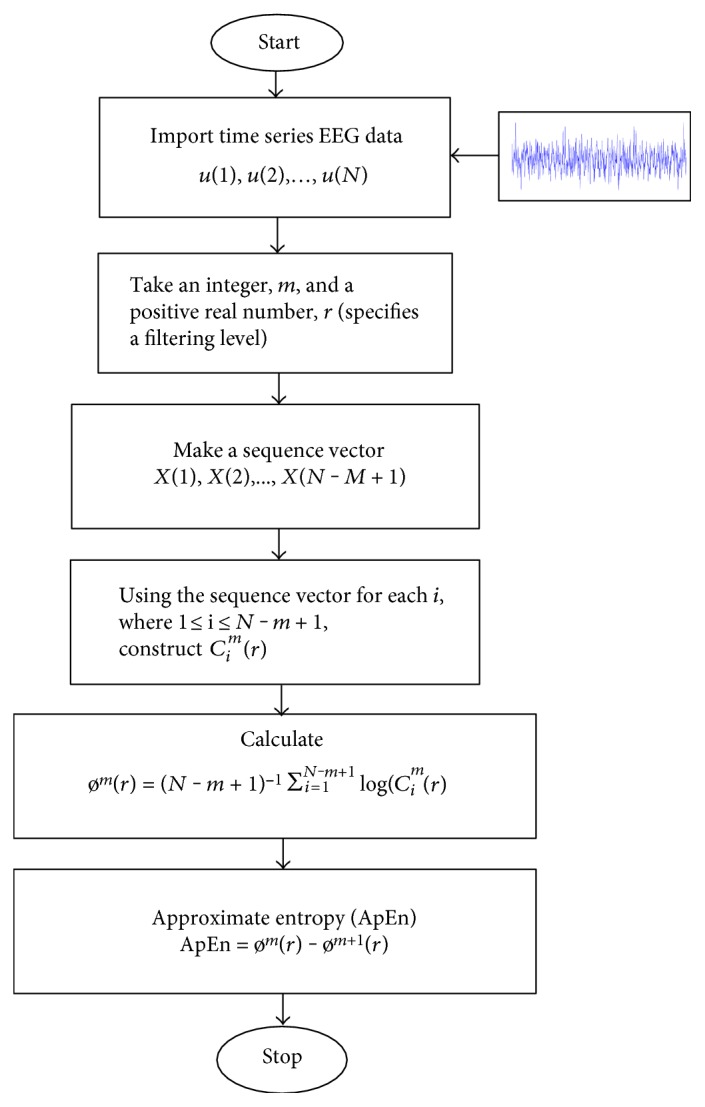
Computational flow diagram for ApEn.

Mathematical procedures of approximate entropy (ApEn) calculation are described in a flow chart [23, 24] in Figure 3.

### 2.3. Standard Deviation (SD) and Standard Error (SE)

The measurements of square root of a variance of random variable, statistical population, any kinds of data set, or probability distribution is known as the standard deviation (SD) which is also known as absolute deviation. The standard deviation can be defined for any distribution with finite first two moments, which can be measured mathematically by using

(9) $$\begin{array}{c} standard\;deviation\;\left(SD\right)=\sqrt{\left(\frac{1}{N}\right)\overset{N}{\underset{n=1}{\sum }}{\left(x_{n}-\mu \right)}^{2}}, \end{array}$$

where *N* is the number of samples in data sets, *x*_*n*_ is the actual value of the *n*th term in data sets, and *μ* is the average value of those data sets. The standard error (SE) is define as the standard deviation (SD) of a sample data set which is the estimation of sample mean based on the population mean. SE is the mean which is calculated using

(10) $$\begin{array}{c} standard\;error\;\left(SE\right)=\frac{standard\;deviation\;\left(SD\right)}{\sqrt{N}}. \end{array}$$

### 2.4. Modified Mean Absolute Value (MMAV)

Mean absolute value (MAV) is the moving average of full-wave rectified data sets which is the measurement of average value by taking the average of absolute value of data sets. So, MMAV is the extension of MAV, in which the individual value is multiplied by weighting function *W*_*n*_ [24] that can be determined by

(11) $$\begin{array}{c} MMAV=\left(\frac{1}{N}\right)\overset{N}{\underset{n=1}{\sum }}\left(w_{n}\times \left|x_{n}\right|\right),\\ where\;w_{n}=\left\{\begin{array}{cc} 1.0, & 0.25N\leq n\leq 0.75N\\ 0.5, & otherwise. \end{array}\right. \end{array}$$

### 2.5. Roll-Off (*R*)

Roll-off is the steepness of a transmission function with frequency, particularly used in signal feature extraction. The roll-off can be defined as the frequency below which 85% of the magnitude distribution of the data sets is intense [24]. It is also a measure of spectral shape which can be written mathematically in

(12) $$\begin{array}{c} R=0.85\times \overset{n/2}{\underset{n=1}{\sum }}\left|x_{n}\right|. \end{array}$$

### 2.6. Zero Crossing (ZC)

Zero crossing (ZC) is the frequency domain features of the data sets which measures the number of times that the amplitude value of data sets crosses the zero *y*-axis [24]. It can be expressed mathematically in

(13) $$\begin{array}{c} ZC=\overset{n}{\underset{n-1}{\sum }}\mathrm{sgn}\;\left(x_{n}\times x_{n-1}\right)\cap \left|x_{n}-x_{n-1}\right|\geq \text{threshold},\\ \mathrm{sgn}\;\left(x\right)=\left\{\begin{array}{cc} 1, & x\geq \text{threshold}\\ 0, & \text{otherwise}. \end{array}\right. \end{array}$$

### 2.7. Regression Analysis

In mathematics, regression analysis is the procedure to find out the mathematical relationship between dependent variables with independent variables. In limited conditions, regression analysis can be used to infer causal relationships between the independent and dependent variables. However, in many applications, especially with small effects or questions of causality based on observational data, regression methods can give misleading results. The function which fits a polynomial regression model by the method of linear least squares is mentioned below.

(14) $$\begin{array}{c} Y=b_{0}+b_{1}x^{1}+b_{2}x^{2}+\cdots +b_{k}x^{k}, \end{array}$$

where *Y* represents predicted outcome value for the polynomial model with regression coefficients *b*_1_ to *b*_*k*_ for the *k*th order polynomial and *Y* intercept *b*_0_.

## 3. Proposed Research Architecture

**Figure 4:**
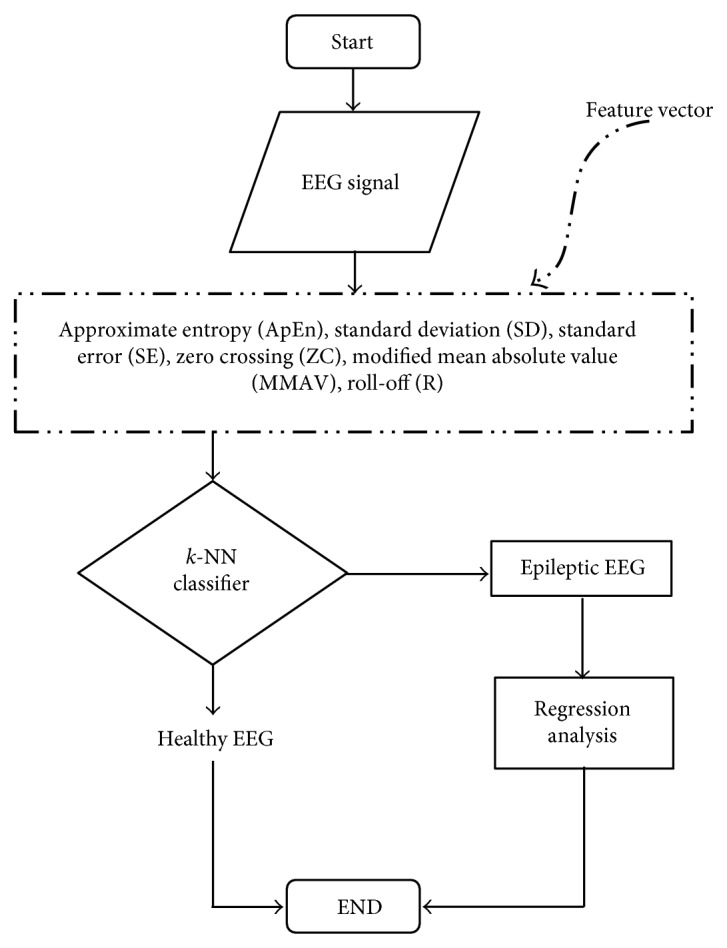
Proposed flow diagram of the research work.

The overall proposed methodology is mentioned in a flow diagram as shown in Figure 4. The epileptic EEG signal is loaded into MATLAB workspace to find out the feature vector of epileptic EEG signal which are approximate entropy (ApEn), standard deviation (SD), standard error (SE), modified mean absolute value (MMAV), roll-off (*R*), and zero crossing (ZC). These feature vectors are classified according to the standard feature vector using *k*-NN classifier. After classification of epilepsy, regression equation is used to find the level of ApEn for different ages of epilepsy from epileptic EEG. High irregular time series EEG signal gives higher value of ApEn and vice-versa. Moreover, higher value of ApEn indicates more irregularity of the epileptic EEG signal. The level of ApEn is increased with the increase of the age of epileptic patients. Finally, error for each interpretation is measured to find out the best fitted equation for the interpretation which is most suitable and optimized regression equation for the prediction.

## 4. Results and Discussions

### 4.1. Classification and Clustering Using *k*-NN

**Figure 5:**
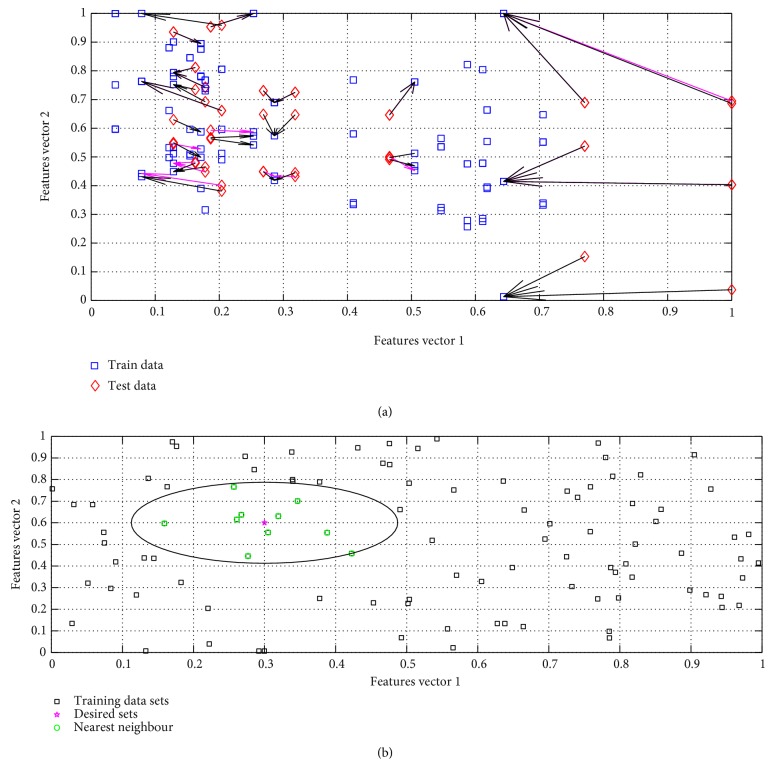
Classification using *k*-NN classifier. (a) Nearest neighbour searching. (b) Clustering with *k*-nearest neighbour.

The epileptic EEG data is processed for the achievement of the feature vector and then a template as mentioned in Table 1 is formed for the train of *k*-NN network. In Table 1, all columns indicate the normalized features set and each row indicates the subject used for the train of network. In Figure 5(a), all the nearest neighbour is determined by the trained *k*-NN network in which all the arrows indicate the nearest neighbour where blue squares indicate the train features set and red diamond are the desired points whose nearest neighbour is our goal. On the other hand, in Figure 5(b), the cluster of the feature vectors (ApEn, MMAV, SD, SE, roll-off, and ZC) is represented using a circle from the classification using *k*-NN classifier. To accomplish the research goal, one desired standard feature point is set as a reference and then the *k*-NN network is trained; its clustering circle is determined around the point of interest. In Figure 5(b), *k* = 10 nearest neighbour is determined inside the circle to find out the close approximation of epileptic EEG signal using feature vectors (ApEn, MMAV, SD, SE, roll-off, and ZC) for that one feature of vector from the normal EEG data (free from the epilepsy) from the patient is required.

### 4.2. Accuracy Analysis of *k*-NN Classifier

**Figure 6:**
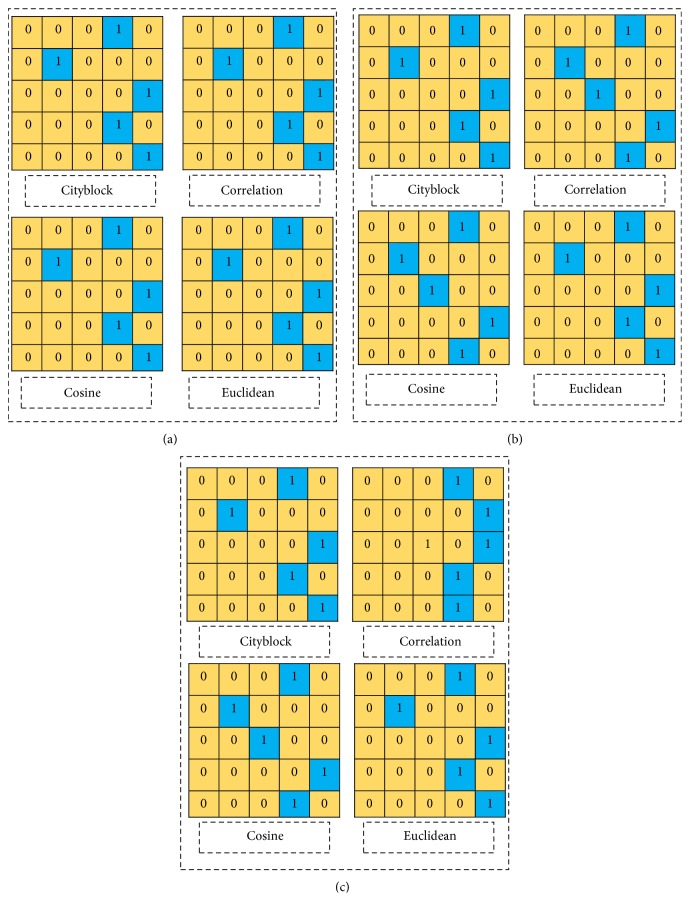
Presentation of confusion matrix for various *k* values, distance types, and classification rules.

In this research work, four distance parameters namely cityblock, correlation, cosine, and Euclidean are used in our analysis and their performance is analysed by considering other parameters keep constant. Similarly, the performance of classifier rule namely nearest neighbour (NN), random neighbour (RN), and smallest neighbour (SN) as well as different *k* values is also analysed keeping corresponding parameters constant. The performance of the *k*-NN classifier which is a confusion matrix is shown in Tables 2 and 3 and Figure 6. From Tables 2 and 3 as well as Figure 6, it is concluded that lower classification rate is found at the “cityblock” when *k* = 1 and the classifier rule is smallest neighbour (SN).

### 4.3. Regression Model for Level of Epilepsy

**Figure 7:**
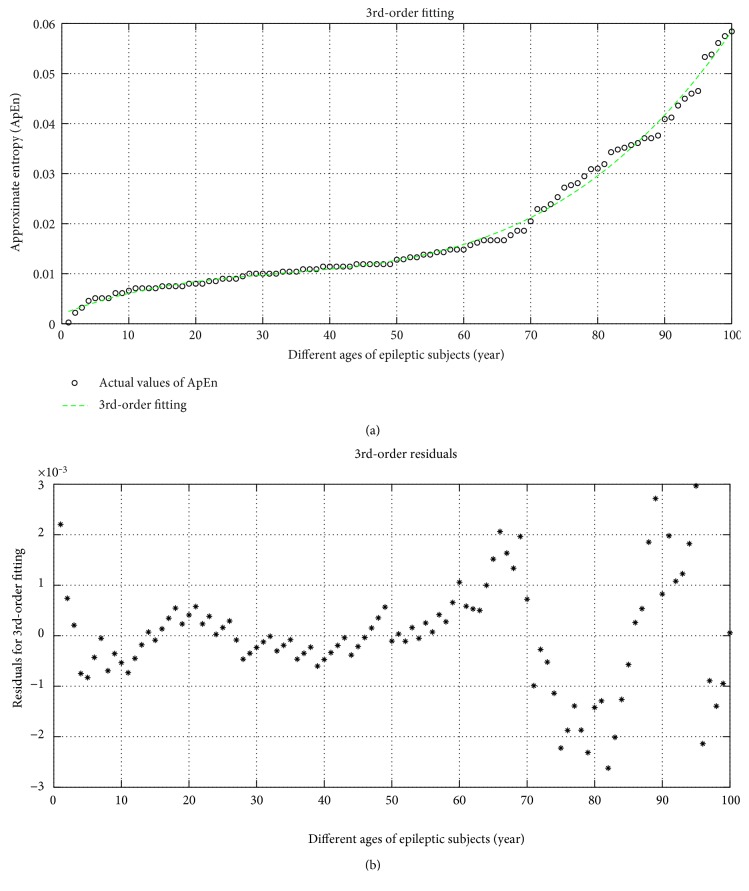
(a) 3rd-order fitting and (b) residual of ApEn with different ages of subjects.

The 3rd-order fittings of the approximate entropy (ApEn) is shown in Figure 7(a). The corresponding regression equation is mentioned in (15). In this equation, if we put the age of the epileptic people, we may be interpreting the degree of randomness of EEG signal.

(15) $$\begin{array}{c} Y_{3rd‐order\;fitting}=1.3277\times 10^{-07}x^{3}-1.2915{\times 10^{-05}x}^{2}+0.00052903x^{1}+0.001932. \end{array}$$

The modification between the predicted value and actual value of the independent value is called the residual which is the measure of accuracy of prediction. The residual of the 3rd-order fitting is shown in Figure 7(b) and its regression equation is (16). From this equation, we may find the error of prediction at any age of the epileptic persons.

(16) $$\begin{array}{c} Y_{3rd‐order\;res}=-8.6358\times 10^{-14}x^{7}+2.9924\times 10^{-11}x^{6}-4.0981\times 10^{-09}x^{5}+2.8173\times 10^{-07}x^{4}-1.0201\times 10^{-05}x^{3}+0.00018623x^{2}-0.0014727x^{1}+0.0032633. \end{array}$$

**Figure 8:**
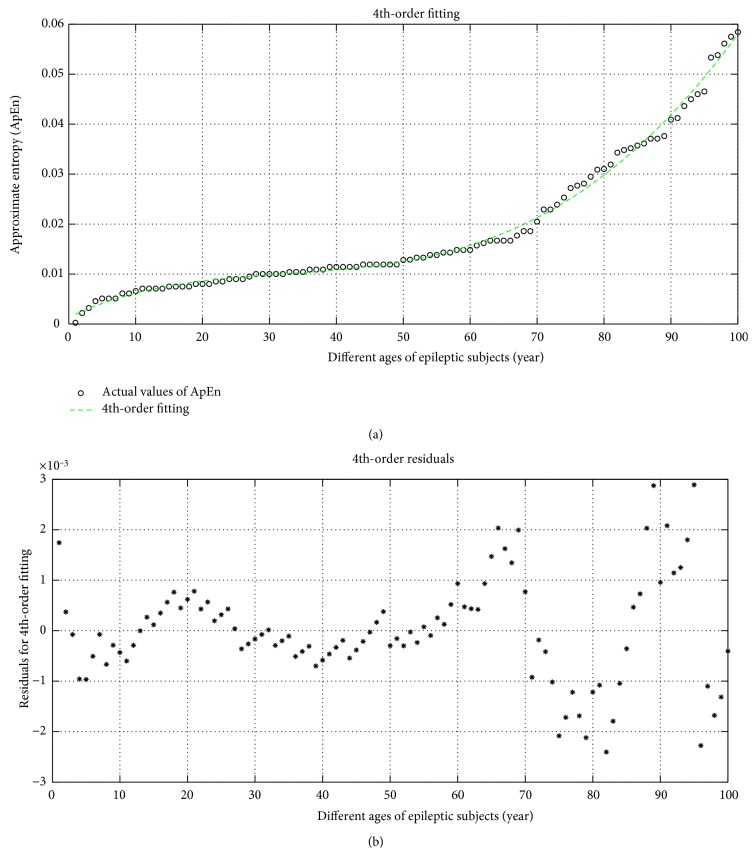
(a) 4th-order fitting and (b) residual of ApEn with different ages of subjects.

In a similar manner, 4th-order fittings of the approximate entropy (ApEn) is shown in Figure 8(a). The corresponding regression equation is mentioned in (17). In this equation, if we put the age of the epileptic people, we may be interpreting the degree of randomness of the EEG signal.

(17) $$\begin{array}{c} Y_{4th‐order\;fitting}=-3.5815\times 10^{-10}x^{4}+2.0511\times 10^{-07}x^{3}-1.7628{\times 10^{-05}x}^{2}+0.00063605x^{1}+0.0013676. \end{array}$$

The residual of 4th-order fitting is shown in Figure 8(b) and its regression equation is (18). From this equation, we may find the error of prediction at any age of the epileptic persons.

(18) $$\begin{array}{c} Y_{4th‐order\;res}=-8.6358\times 10^{-14}x^{7}+2.9924\times 10^{-11}x^{6}-4.0981\times 10^{-09}x^{5}+2.8173\times 10^{-07}x^{4}-1.0129\times 10^{-05}x^{3}+0.00018151x^{2}-0.0013657x^{1}+0.0026987. \end{array}$$

### 4.4. Error Analysis of Prediction

In Table 4, the error of prediction is shown where the accuracy of prediction (interpretation) is more in the 3rd-order fitting. The 1st-order fitting is a liner fitting like *y* = *mx* + *c* which has more error probability and also it has a larger value of residual than other types of fitting of ApEn. From the table, it is noticed that the increase of order of fitting may reduce the error probability, but after the 3rd-order fitting, the error probability as well as the computational complexity is increased. Hence, optimum prediction equation for the epileptic seizure is the 3rd-order which has less computational complexity and less error probability than the 4th-order fitting. Form the table, it is also remarkable that at the smaller age of the epileptic people, the prediction error is more because at the increasing ages of the epileptic persons the EEG (epileptic) is more severe.

## 5. Conclusions

The electrophysiological activity of the brain called EEG signal can analyze for the prediction and diagnosis of epilepsy of the living animals. The epileptic EEG signal is more and more random and this EEG containing epilepsy is not suitable for the perfect brain-computer interface (BCI) paradigms. Hence, prediction of epilepsy is a vital issue in the modern biomedical field of research. For the prediction of epilepsy, a statistical approach was explained in this manuscript. In our research, the epileptic EEG signals for different aged epileptic subjects was analyzed and one of the vital features Approximate entropy (ApEn) was measured which was the indicator of randomness of any time domain signal. The regression equation of ApEn with respect to different ages of the epileptic persons may help the BCI researchers or the neural researcher to predict the randomness, namely, level of epilepsy corresponding to different ages. This may help the clinical person to provide the treatment of the epileptic person after finding the level of randomness.

## Figures and Tables

**Table 1:** Training and testing template of feature vectors of epileptic EEG data.

Subjects of train or test	Normalized features					
	ApEn	SD	SE	MMAV	Roll-off	ZC
S_1_	0.17123	0.87575	0.87574	0.78181	0.77964	0.39014
S_2_	0.20376	0.80575	0.80574	0.51230	0.49013	0.59653
⋮	⋮	⋮	⋮	⋮	⋮	⋮
S_20_	0.61815	0.66366	0.66367	0.39526	0.39025	0.55392

**Table 2:** Percentage of accuracy due to variation of nearest number *k* and other parameters kept constant.

Distance types	*k* = 1 and nearest neighbour (NN)		*k* = 2 and nearest neighbour (NN)		*k* = 3 and nearest neighbour (NN)	
	Accuracy	Confusion matrix	Accuracy	Confusion matrix	Accuracy	Confusion matrix
“Cityblock”	60%	Figure 6(a)	60%	Figure 6(a)	60%	Figure 6(a)
“Correlation”	60%		60%		60%	
“Cosine”	60%		60%		60%	
“Euclidean”	60%		60%		60%	

**Table 3:** Percentage of accuracy due to variation of classification rule other parameters kept constant.

Distance types	*k* = 1 and nearest neighbour (NN)		*k* = 1 and random neighbour (RN)		*k* = 1 and smallest neighbour (SN)	
	Accuracy	Confusion matrix	Accuracy	Confusion matrix	Accuracy	Confusion matrix
“Cityblock”	60%	Figure 6(a)	40%	Figure 6(b)	20%	Figure 6(c)
“Correlation”	60%		60%		60%	
“Cosine”	60%		40%		40%	
“Euclidean”	60%		60%		60%	

**Table 4:** Error (% deviation) calculation for different orders of fitting and for different test values (age) of subjects.

Order of fittings	Value of ApEn (20 years of subject)		Value of ApEn (40 years of subject)		Value of ApEn (60 years of subject)		Value of ApEn (80 years of subject)	
	Actual	Interpreted	Actual	Interpreted	Actual	Interpreted	Actual	Interpreted
3rd order	0.008	0.00841	0.0114	0.0109	0.0148	0.0159	0.031	0.0310
	Error = 5.13%		Error = 4.39%		Error = 7.43%		Error = 2.51%	
4th order	0.008	0.00862	0.0114	0.0108	0.0148	0.0157	0.031	0.0312
	Error = 7.75%		Error = 5.26%		Error = 6.08%		Error = 2.52%	
